# Clinicopathological correlation of aspartate aminotransferase-to-platelet ratio (APRI) and aspartate aminotransferase-to-alanine aminotransferase ratio (AAR) with survival in esophageal carcinoma patients: a retrospective cohort analysis of 951 patients

**DOI:** 10.1097/MS9.0000000000000311

**Published:** 2023-03-14

**Authors:** Muhammad Talha Zafar, Beenish Fatima Zia, Saleha Rashid Khalid, Jharna Bai, Zahid Ali Memon, Zaka Ullah Jan, Sarosh Khan Jadoon, Noman Ahmed Khan, Diksha Kajal, Farukh Ali, Tooba Ahmed Kirmani, Muhammad Sohaib Asghar

**Affiliations:** a DHQ Hospital, Jauharabad; b FMH College of Medicine and Dentistry, Lahore, Punjab; c Shaheed Mohtarma Benazir Bhutto Medical University (SMBBMU), Larkana, Sindh; d Civil Hospital; e Jinnah Medical and Dental College; f Dow University Hospital–Ojha Campus, Dow University of Health Sciences, Karachi; g Ghulam Muhammad Mahar Medical College, Sukkur; h Khyber Teaching Hospital, Peshawar; i CMH Muzaffarabad, Azad Kashmir, Pakistan

**Keywords:** malignancy, esophagus, histopathology, survival, mortality

## Abstract

Esophageal cancer is the eighth most prevalent cancer globally. Previously, several biomarkers have been used to predict the prognosis, although with variable reliability. Interestingly, it is noted that changes in liver function tests levels before and after neoadjuvant treatment are predictive in terms of cancer recurrence.

**Objectives:**

The objectives of the current study were to associate novel markers, including aspartate aminotransferase-to-platelet ratio (APRI) and aspartate aminotransferase-to-alanine aminotransferase ratio (AAR) with survival in esophageal malignancy.

**Materials and Methods:**

A retrospective study in a tertiary care hospital (single-center) included 951 patients having diagnosed esophageal carcinoma of any age group.

**Results:**

The median (interquartile range) age of study participants were 50 (38–60) years, including 43% males and 57% female patients, while the median (interquartile range) levels of AAR and APRI were 0.97 (0.81–1.25) and 0.19 (0.13–0.29), respectively. AAR was found to be higher in dysphagia for solids only and dysphagia for both liquids and solids rather than liquids only (*P*=0.002), while other associations included well-differentiated tumor grade (*P*=0.011), finding of esophageal stricture on esophagogastroduodenoscopy (*P*=0.015), and characteristic of mass on computerized tomography scan being both circumferential and mural (*P*=0.005). APRI was found to be higher in adenocarcinoma (*P*=0.038), and finding of circumferential±ulcerated mass on esophagogastroduodenoscopy (*P*<0.001). On survival analysis, adenocarcinoma (*P*<0.001), luminal narrowing (*P*=0.002), AAR greater than 1.0 (*P*=0.006), and APRI greater than 0.2 (*P*=0.007) were found to be poor survival predictors. On Cox proportional hazards regression, APRI was found to be more associated with poor survival than AAR (Hazard ratio: 1.682, 1.208–2.340, *P*=0.002).

**Conclusion:**

This study correlated clinical and pathological features of esophageal malignancy with noninvasive markers of hepatic function.

## Introduction

Highlights

- Changes in liver function tests levels can be predictive in terms of cancer prognosis.
- Our study correlated clinical and pathological features of esophageal malignancy with noninvasive markers of hepatic function.
- These markers were already established in chronic liver disease prognosis.

Esophageal cancer is ranked as the eighth most prevalent cancer worldwide^1^. It is also known to be the sixth leading cause of mortality from cancer^2^. On the basis of histology, esophageal squamous cell carcinoma is more prevalent^3^. Available treatment options include surgery only, or chemotherapy along with surgery, and radiotherapy^3^. Esophageal cancer is often diagnosed in its advanced stages, so surgical resection is possible in only 30–40% of cases^4^. The 5-year survival rate is 20% after surgery in such patients^5^. Previously, several biomarkers such as Squamous cell carcinoma, cytokeratin 19 fragment, and Carcinoembryonic antigen have been used to predict the prognosis^6^. Though, their reliability is still doubted.

Interestingly, it is noted that changes in liver function tests levels before and after neoadjuvant treatment are predictive in terms of cancer recurrence^3^. Precisely, Serum Alanine aminotransferase (ALT), aspartate aminotransferase (AST), and AST/ALT ratio (AAR) are of use. The AST and ALT are the transaminases present in circulation and are important indicators of liver dysfunction^7^. There is a possibility that the production of AST and ALT can be altered because of cancer-associated changes in metabolism. Moreover, the AST/ALT ratio has been linked with survival outcomes in the case of many cancers^7^. Second, activation of platelets also have a role to play in tumorigenesis and its progression^8^.

The aspartate aminotransferase-to-platelet ratio (APRI) is the score that is calculated from the AST and platelet ratio^9^. It is approved as a reliable marker for assessing liver function reserve as well as the prognosis of hepatocellular carcinoma^9^. However, limited data exists regarding the correlation of esophageal cancer with the AST/ALT ratio and APRI.

Therefore, to broaden our horizons, in our study, we attempted to find any association of AAR and APRI with survival outcomes in patients with esophageal cancer.

## Materials and methods

This retrospective study was conducted at the Department of Gastroenterology at a tertiary care hospital, Karachi, Pakistan. An ethical review was obtained by the IRB, and research protocol was registered with the local registry of Dow University Hospital (UIN: IRB/DUH/2021/789). Medical records of all patients admitted to our hospital from 1 January 2011 to 30 December 2020, with a diagnosis of esophageal carcinoma having a mass lesion or luminal narrowing were included. In-situ carcinoma on histopathology was excluded (*n*=23). Those patients have hepatic metastasis were also excluded from the analysis (*n*=35). The nonprobability consecutive sampling method was used to recruit the patients.

Data regarding the patient’s age, sex, ethnic and regional affiliation, comorbidities, occupation, and symptomatology were collected from the individual medical records. Clinical data included histopathology, esophagogastroduodenoscopy (EGD), computerized tomography (CT) scan, and laboratory markers. The results of ALT, AST, AST/ALT ratio (AAR), platelet counts, and APRI were collected on admission.

### Statistical analysis

Data were analyzed via SPSS version 25.0 (IBM Corp.). Median, interquartile range (IQR), frequency, and relative percentages were reported for descriptive variables. Nonparametric tests like Mann–Whitney *U* and Kruskal–Wallis *H* tests were used for quantitative variables due to their nonuniform distribution as determined by the Shapiro–Wilk test. Post-hoc Donn–Bonferroni was applied to significant results. Kaplan–Meier curve analysis was carried out to associate poor survival predictors. Cox proportional hazard models were used to calculate the survival probability. A *P* value of less than 0.05 was considered statistically significant (two-tailed). The manuscript is reported according to STROCSS guidelines^10^.

## Results

**Figure 1:**
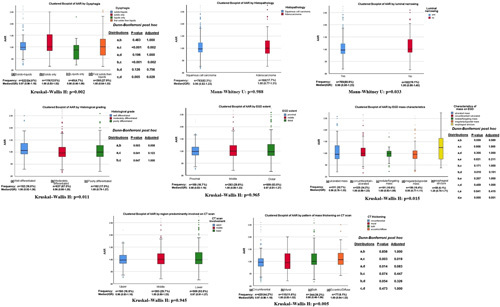
Correlation factors of aspartate aminotransferase-to-alanine aminotransferase ratio with features of esophageal cancer.

The median (IQR) age of study participants was 50 (38–60) years, while the median (IQR) levels of AAR and APRI were 0.97 (0.81–1.25) and 0.19 (0.13–0.29), respectively. The optimal cutoff points selected using ROC curve analysis for AAR was greater than 1.0, and for APRI was found greater than 0.2. The male-to-female ratio was 1 : 1.3 as shown in Table 1. Dysphagia followed by weight loss were the most predominant initial presenting symptoms. The progression of dysphagia was common for either solids and liquids together (55%) or solids initially progressing to liquids (28%). AAR was found higher in dysphagia for solids only and dysphagia for both liquids and solids rather than liquids only (*P*=0.002), while other associations included well-differentiated tumor grade (*P*=0.011), finding of esophageal stricture on EGD (*P*=0.015), and characteristic of mass on CT scan being both circumferential and mural (*P*=0.005) as shown in Figure 1.

**Table 1:** Baseline, sociodemographic, and clinical characteristics of included esophageal cancer patients (*n*=951)

Age	Median (IQR)	50 (38–60), *n* (%)
Sex	Male	412 (43.3)
	Female	539 (56.7)
Initial symptoms	Fever	24 (2.5)
	Weight loss	183 (19.2)
	Upper gastrointestinal bleed	44 (4.6)
	Retrosternal burning	60 (6.3)
	Acid reflux	21 (2.2)
	Dysphagia	308 (32.4)
	Vomiting (projectile)	180 (18.9)
	Vomiting (nonprojectile)	39 (4.1)
	Odynophagia	42 (4.4)
	Anorexia	4 (0.4)
	Sticking of foods	7 (0.7)
	None	39 (4.1)
Progression of dysphagia	Solids+liquids together	522 (54.9)
	Solids only	119 (12.5)
	Liquids only	45 (4.7)
	First solids then liquids	265 (27.9)
Histopathology	Squamous cell carcinoma	783 (82.3)
	Adenocarcinoma	168 (17.7)
Degree of differentiation	Well differentiation	152 (16.0)
	Moderate differentiation	637 (67.0)
	Poor differentiation	162 (17.0)
Local extension on EGD	Proximal	159 (16.7)
	Middle	283 (29.8)
	Distal	509 (53.5)
Mass lesion characteristics on EGD	Ulcerated mass	311 (32.7)
	Circumferential±ulcerated	325 (34.2)
	nodular/fungating mass	101 (10.6)
	irregular/polypoidal mass	156 (16.4)
	esophageal stricture	58 (6.1)
Luminal narrowing	Yes	769 (80.9)
	No	182 (19.1)
Predominant region involved on CT scan	Upper	160 (16.8)
	Middle	282 (29.7)
	Lower	509 (53.5)
Pattern of mass thickening on CT scan	Circumferential	420 (44.2)
	Mural	110 (11.6)
	Both circumferential+mural	344 (36.2)
	Eccentric or diffuse	77 (8.1)
Survival on follow-up	Alive	451 (47.4)
	Death	500 (50.0)

CT, computerized tomography; EGD, esophagogastrodudenoscopy.

**Figure 2:**
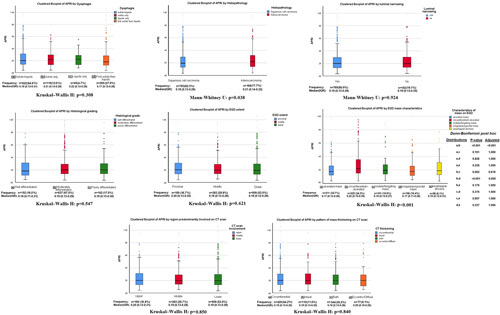
Correlation factors of aspartate aminotransferase-to-platelet ratio with features of esophageal cancer.

**Figure 3:**
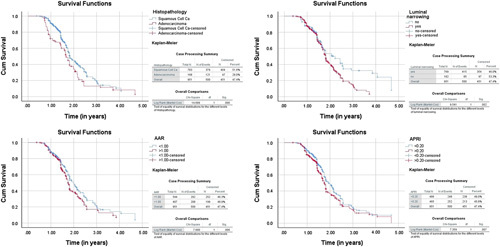
Kaplan–Meier curves for studied prognostic factors of esophageal cancer.

Squamous cell carcinoma was prevalent in our study population (82%), and moderately differentiated features were more likely on histopathology (two-thirds). The lower one-third of the esophagus was commonly involved, which was evaluated both on EGD as well as CT scan. Luminal narrowing was exhibited by 81% of the individuals. The most frequent characteristics of mass lesions were ulcerated on EGD and circumferential on CT scan. APRI was found higher in adenocarcinoma (*P*=0.038), and finding of circumferential±ulcerated mass on EGD (*P*<0.001) as shown in Figure 2. On survival analysis, adenocarcinoma (*P*<0.001), luminal narrowing (*P*=0.002), AAR greater than 1.0 (*P*=0.006), and APRI greater than 0.2 (*P*=0.007) were found poor survival predictors (Fig. 3). On Cox proportional hazards regression, APRI was found associated with poor survival with Hazard ratio: 1.682 (1.208–2.340), *P*=0.002, but AAR was not found significant after adjustment.

## Discussion

In esophageal carcinoma, there is a necessity to identify more specific biomarkers for better estimation of the prognosis and timely diagnosis. As mentioned earlier, AAR and APRI play a part in carcinogenesis. These tests can be done routinely, and their reporting takes less time. Second, they are noninvasive. These characteristics make them suitable for predicting prognosis earlier in esophageal cancer patients.

In previous studies, the AST/ALT ratio (AAR) has been linked with certain malignancies, such as breast cancer, gastric cancer, and liver cancer, etc. The ALT and AST also serve the purpose of enzymes to form products in gluconeogenesis and amino acid metabolic processes. They both catalyze the transfer of amino groups being specific markers of liver dysfunction^3^. On cellular level, AST is mainly present in mitochondria and is found in various organs (liver, heart, kidney, brain, and skeletal muscle) while ALT exists only in the cytoplasm of a hepatocyte. Technically, serum AST should be greater than serum ALT because of the hepatic distribution of 2.5 : 1 of AST/ALT. But as AST is removed quickly (*t*_1/2_=18 h) by the liver sinusoids in contrast to AST (*t*_1/2_=36 h) so their serum levels are the same. This ratio remains unchanged in healthy people^7^.

In the literature, we found that previous research was utilizing ALT/AST ratio for prognosis, which is the inverse of AAR used in the current study. Huang *et al.* 3 noticed that esophageal carcinoma patients who had increased reverse AAR levels demonstrated remarkably better prognosis in contrast to those who had decreased reverse AAR levels. Similarly, in our study, higher AAR was found predictive of survival, which corresponds with the findings of Huang *et al* 3. Dong Tian *et al.* 11 also found out that higher reverse AAR levels indicate a good prognosis, as they affect proinflammatory mediators, which are an active part of carcinogenesis, tumor invasion, and metastases. And also that reverse AAR levels correlate with greater risk of lymph node metastasis in esophageal squamous cell carcinoma.

Kimm and colleagues witnessed that both AST and ALT levels individually, as well as the AST/ALT ratio, have an association with an increased risk of esophageal cancer. And also that alcohol intake and AAR levels are independent risk factors for the occurrence of esophageal cancer^12^. When it comes to symptoms, dysphagia is common in esophageal cancer patients^13^. Specifically, if it progressed rapidly within weeks or months along with significant weight loss^14^. However, in our study, we found the higher AAR levels in dysphagia for solids only and dysphagia for both liquids and solids rather than liquids only, indicating link between higher AAR levels and the progression of disease in esophageal cancer patients.

According to the available literature, platelet activation is involved in the progression of cancer. The worse clinical outcome is observed due to thrombocytosis in patients with breast cancer, pancreatic cancer, lung cancer, and colorectal cancer^15^. The significance of how predictive the APRI score is in case of liver cirrhosis and fibrosis in hepatitis B virus patients has already been established^16^. Kelvin Allenson *et al.* 17 showed that APRI can predict mortality in case of hepatocellular carcinoma on its own rather than depending on Model for End-Stage Liver disease score. One study done in Nepal revealed that the APRI score is able to diagnose esophageal varices in liver cirrhosis patients, at places where endoscopic facilities are not easily accessible^18^. Hung-Hsu Hung *et al.* 19 demonstrated that APRI is helpful to assess the extent of hepatic fibrosis; for evaluating liver functional reserve, and survival rate in patients with hepatitis B virus-related hepatocellular carcinoma. However, there is not much research done on the role of APRI in predicting prognosis in esophageal cancer. In our study, we found out that patients with esophageal cancer who have higher APRI scores had certain clinicopathological correlations and prognostic implications.

This study is a single-center analysis, hence limiting the generalizability of the findings. Although hepatic metastasis patients were excluded, there are other confounding factors like hepatic steatosis that remain undocumented in the study and could have influenced the hepatic marker derangements. There was limited data available on mode of treatment and distant metastasis, hence we cannot assimilate the overall outcomes with those variables. The stages of cancer cases included in this study were not specified. There was also a lack of cancer-specific survival data so outcomes based on the overall cause of death could not specified to disease-specific mortality.

## Conclusions

Our study correlated clinical and pathological features of esophageal malignancy with noninvasive markers of hepatic function. Further studies can be able to find out the longitudinal association of these markers with the clinical outcomes, as data did not account for follow-up with serial evaluation of these markers.

## Ethical approval

Ethical approval was obtained in this study from Institutional Review Board.

## Consent

Consent to participate from the patients was waived and not required due to retrospective nature of the data collection.

## Sources of funding

No funding required for the study.

## Author contribution

T.A.K., and M.S.A.: conceived the idea. B.F.Z., S.R.K., M.T.Z., S.K.J., and N.A.K.: collected the data. D.K. and J.B.: analyzed and interpreted the data. F.A. and M.S.A.: did write up of the manuscript. Z.U.J., Z.A.M., and M.S.A. reviewed and revised the manuscript for intellectual content critically. All authors approved the final version of the manuscript.

## Conflict of interest disclosure

The authors have no conflicts of interest to declare.

## Research registration unique identifying number (UIN)

1. Name of the registry: Dow University Hospital.
2. Unique identifying number or registration ID: (UIN/DUH/2021/789).
3. Hyperlink to your specific registration (must be publicly accessible and will be checked): https://www.duhs.edu.pk/departments/research/downloads/IRB%20Form-20150410.doc

## Guarantor

Muhammad Sohaib Asghar.

## Data availability statement

All data will be made available on a reasonable request to the corresponding author.

## Provenance and peer review

Externally peer reviewed, not commissioned.
